# Effect of Qihuang Decoction Combined with Enteral Nutrition on Postoperative Gastric Cancer of Nutrition and Immune Function

**DOI:** 10.1155/2020/1795107

**Published:** 2020-02-29

**Authors:** Qing-sheng Yu, Zhou Zheng, Hui Peng, Yi Shen, Ju-da Liu, Fu-hai Zhou

**Affiliations:** ^1^Department of General Surgery, The First Affiliated Hospital of Anhui University of Chinese Medicine, Hefei, China; ^2^Institute of Chinese Medicine Surgery, Anhui University of Traditional Chinese Medicine, Hefei, China

## Abstract

**Objective:**

Early nutritional support in patients with gastric cancer can improve their nutritional status, but the impact on immune function has not been confirmed. This study aimed to analyze the effects of Qihuang decoction combined with enteral nutrition on nutrition and the immune function of postoperative gastric cancer.

**Methods:**

120 patients with postoperative gastric cancer in the study group and 117 in the control group were selected as the study subjects from our hospital at random. Indications of nutrition and immune and the rates of complications were compared the day before surgery and 1, 3, 7, and 14 days after surgery.

**Results:**

Indications of nutrition except hemoglobin (HB) in the study group were significantly higher than those before operation and the albumin (ALB) and prealbumin (TP) were significantly increased 7 and 14 days after surgery (*P* < 0.001 and *P* < 0.001 and *P* < 0.001 and *P* < 0.001 and *P* < 0.001 and *P* < 0.001 and *P* < 0.001 and *P* < 0.001 and *P* < 0.001 and *P* < 0.001 and *P* < 0.001 and *P* < 0.001 and *P* < 0.001 and *P* < 0.001 and *P* < 0.001 and *P* < 0.001 and *P* < 0.001 and *P* < 0.001 and *P* < 0.001 and *P* < 0.001 and *P* < 0.001 and *P* < 0.001 and *P* < 0.001 and *P* < 0.001 versus *P* < 0.001 and *P* < 0.001) and the protein (PA) 3, 7, and 14 days after surgery (*P*=0.011, *P*=0.002, and *P*=0.022) in the study group compared to those in the control group. Cellular and humoral immunity indications in the study group are significantly higher than those before operation compared to those in the control group, and the CD3^+^, CD4^+^, and CD4^+^/CD8^+^ were significantly increased 7 and 14 days after surgery (*P*=0.027 and *P* < 0.001 versus *P*=0.008 and *P* < 0.001 versus *P*=0.010 and *P* < 0.001) and IgA, IgG, and IgM 3, 7, and 14 days after surgery in the study group (*P* < 0.001, *P* < 0.001, and *P* < 0.001 versus *P* < 0.001, *P* < 0.002, and *P* < 0.001 versus *P* < 0.001, *P* < 0.001, and *P* < 0.001). The complications such as abdominal, lung, wound, and urinary infection were also significantly decreased (*P*^*χ*^2^^=0.017; *P* < 0.001 and *P* < 0.001 versus *P* < 0.001 and *P* < 0.001) and the protein (PA) 3, 7, and 14 days after surgery (*P*=0.011, *P*=0.002, and *P*=0.022) in the study group compared to those in the control group. Cellular and humoral immunity indications in the study group are significantly higher than those before operation compared to those in the control group, and the CD3^+^, CD4^+^, and CD4^+^/CD8^+^ were significantly increased 7 and 14 days after surgery (*P*=0.027 and *P* < 0.001 versus *P*=0.008 and *P* < 0.001 versus *P*=0.010 and *P* < 0.001) and IgA, IgG, and IgM 3, 7, and 14 days after surgery in the study group (*P* < 0.001, *P* < 0.001, and *P* < 0.001 versus *P* < 0.001, *P* < 0.002, and *P* < 0.001 versus *P* < 0.001, *P* < 0.001, and *P* < 0.001). The complications such as abdominal, lung, wound, and urinary infection were also significantly decreased (*P*^*χ*^2^^=0.017; *P* < 0.001 and *P* < 0.001 versus *P* < 0.001 and *P* < 0.001) and the protein (PA) 3, 7, and 14 days after surgery (*P*=0.011, *P*=0.002, and *P*=0.022) in the study group compared to those in the control group. Cellular and humoral immunity indications in the study group are significantly higher than those before operation compared to those in the control group, and the CD3^+^, CD4^+^, and CD4^+^/CD8^+^ were significantly increased 7 and 14 days after surgery (*P*=0.027 and *P* < 0.001 versus *P*=0.008 and *P* < 0.001 versus *P*=0.010 and *P* < 0.001) and IgA, IgG, and IgM 3, 7, and 14 days after surgery in the study group (*P* < 0.001, *P* < 0.001, and *P* < 0.001 versus *P* < 0.001, *P* < 0.002, and *P* < 0.001 versus *P* < 0.001, *P* < 0.001, and *P* < 0.001). The complications such as abdominal, lung, wound, and urinary infection were also significantly decreased (*P*^*χ*^2^^=0.017; *P* < 0.001 and *P* < 0.001 versus *P* < 0.001 and *P* < 0.001) and the protein (PA) 3, 7, and 14 days after surgery (*P*=0.011, *P*=0.002, and *P*=0.022) in the study group compared to those in the control group. Cellular and humoral immunity indications in the study group are significantly higher than those before operation compared to those in the control group, and the CD3^+^, CD4^+^, and CD4^+^/CD8^+^ were significantly increased 7 and 14 days after surgery (*P*=0.027 and *P* < 0.001 versus *P*=0.008 and *P* < 0.001 versus *P*=0.010 and *P* < 0.001) and IgA, IgG, and IgM 3, 7, and 14 days after surgery in the study group (*P* < 0.001, *P* < 0.001, and *P* < 0.001 versus *P* < 0.001, *P* < 0.002, and *P* < 0.001 versus *P* < 0.001, *P* < 0.001, and *P* < 0.001). The complications such as abdominal, lung, wound, and urinary infection were also significantly decreased (*P*^*χ*^2^^=0.017; *P*^*χ*^2^^=0.036; *P*^*χ*^2^^=0.041; *P*^*χ*^2^^=0.004).

**Conclusions:**

Qihuang decoction combined with enteral nutrition can promote the absorption of enteral nutrition with improving the immune and reducing complications of infection.

## 1. Introduction

Gastric cancer is the third leading cause of cancer death in the world. Among the world's geographical regions, the highest incidence and mortality of gastric cancer are in Northeast Asian countries, including China, Japan, and South Korea, accounting for more than half of the world's total [1, 2]. Owing to the characteristics of vigorous proliferation ability and autonomy of malignant tumor and the stress of preoperative fasting, surgery, and anesthesia, patients with gastric cancer have had cachexia such as marasmus, anemia, and other diseases by increasing the catabolism of the body and causing the body to be in negative nitrogen balance [3, 4]. Malnutrition not only is not conducive to wound healing but also increases the incidence of complications and mortality in patients. Moreover, inhibition of immunity leads to metastasis and recurrence of tumors, so early nutritional support after gastric cancer surgery is particularly significant [5–7].

At present, the main methods of postoperative nutrition are parenteral nutrition (PN) and enteral nutrition (EN). However, PN can provide glucose, amino acid, and other nutrients for organs and tissues for postoperative patients. Owing to long-term digestive tract disposition, intestinal microecology is prone to disorder or bacterial translocate through atrophy of intestinal mucosa and disruption of the intestinal barrier, which not only increase enterogenic infection rate but also trigger systemic inflammatory response [8, 9]. In contrast, EN can promote the growth and repair of damaged intestinal mucosal cells, maintain the balance and growth of the inherent flora in the gastrointestinal mucosa, and stimulate the secretion and releases of various related hormones so that it helps the recovery of the gastrointestinal motility [10–12]. However, gastroreflux aspiration pneumonia and short-term gastrointestinal symptoms occur frequently, such as diarrhea which may result in loss of nutrients and imbalance of water, electrolyte, acid, and base [13, 14]. Since the 1990s, some researchers have tried to promote the recovery of the intestinal mucosa by adding special nutrients such as arginine, glutamine, *ω*-fatty acids, nucleosides, and nucleotides to the standard enteric nutrient solution. We notice that the immunity of patients was improved to some extent, but the nutritional status of patients did not have an obvious advantage [15].

Our previous animal experiments showed that Qihuang decoction not only promoted the recovery of intestinal immune barrier in rats after gastrectomy but also improved the mechanical barrier of intestinal mucosa [16]. This study is to discuss the nutritional status, immune function, gastrointestinal function recovery, and complications of postoperative patients with gastric cancer in the early stage after gastric cancer in our hospital through intranasal feeding of Qihuang decoction combined with enteral nutrition emulsion.

## 2. Patients and Methods

### 2.1. Ethics Statement

The study was approved by the Ethics Committee of the First Affiliated Hospital of Anhui University of Traditional Chinese Medicine and complied with the Helsinki Declaration. All participants gave written informed consent before collecting data.

### 2.2. Patient Population

A total of 237 patients were ultimately selected from the first affiliated hospital of Anhui University of Traditional Chinese Medicine on January 1, 2015, and December 31, 2018, for gastric cancer surgery, including 120 patients in the study group and 117 patients in the control group. Diagnostic criteria were in line with the relevant standards for the diagnosis of gastric cancer diagnostic criteria issued in Japan in 2010 [17]. Inclusion criteria were as follows: patients with gastric cancer diagnosed by gastroscopy and pathology were selected for surgical treatment and patients who had not used chemotherapy for half a year. Exclusion criteria were as follows: patients who had have gastrointestinal dysfunction, abnormal liver function, intestinal absorption, metabolic disorders, immune dysfunction, or digestive system diseases; patients with severe malnutrition (BMI < 18 kg/m^2^); pregnant and lactating women; patients with severe accompanying diseases such as chronic cardiopulmonary insufficiency and chronic renal failure; and patients who had have a history of cerebral infarction less than 6 months, and radical surgery could not be performed owing to patients with distant metastases found during surgery.

### 2.3. Allocation to Groups

**Figure 1:**
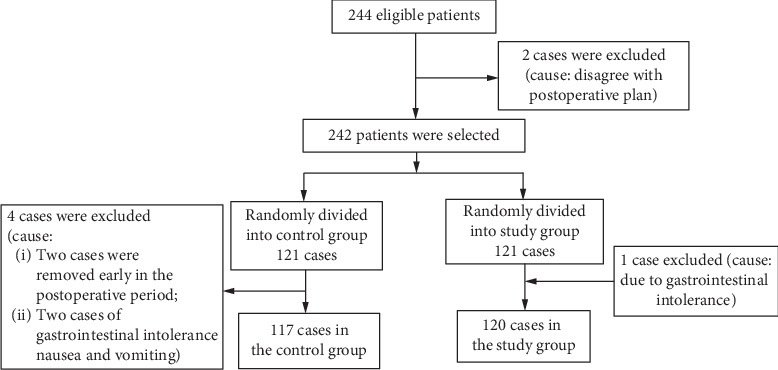
Diagram showing the flow of participants' enrollment.

The 244 patients who were initially recruited were randomly divided into the study group and control group according to 1 : 1, and random numbers (range 0 to 1) were generated for 244 using SPSS21.0 software. Then, the rank was compiled, taking 1 to 122 as the study group and 123 to 244 as the control group, the 001–244 marked strips were placed in an opaque envelope, and the patients randomly selected the strips. Then, we grouped the extracted strips digitally. Sealed envelopes are supervised by a specially assigned person, and patients and medical staff are completely unaware of the data and research (Figure 1).

### 2.4. Intervention

#### 2.4.1. Preoperative Preparation

Both groups of patients performed the same preparation before surgery. Fasting water and diet 24 hours before surgery and oral catharsis medication to diarrhea 8 hours before surgery were performed by all patients for preoperative bowel preparation. 30 minutes before surgery, the second-generation cephalosporin was used to prevent postoperative abdominal infection, and a jejunal nutrient tube (trade name: Fuerkai Nasogastric tube, standard number: YZB/Su0943-2014; the manufacturer: Nutricia Pharmaceuticals Wuxi Co., Ltd.) was inserted into the side hole of the lowermost part of the stomach tube. The surface of the two tubes is coated with paraffin oil. When the tube is intubated, the patient took a deep breath and swallows normally until the two tubes are inserted into the stomach cavity through the patient's nostrils (the depth is 50 to 60 cm) and the syringe is pumped with gastric juice out. The stomach tube is fixed with a tether and the jejunal nutrition tube is fixed with a tape.

#### 2.4.2. Postoperative Treatment

*(1) Control Group*. Enteral nutrition emulsion was offered (TPF 500 ml approval number: National Pharmaceutical Standard H20040188, Ruixian, 28 g of protein, 29 g of fat, 94 g of carbohydrate, 10 g of dietary fiber, various minerals and vitamins, and total energy supply of 750 kcal); 0.9% sodium chloride 100 ml was given at 16 h after operation, and mixed suspension that contains 250 ml TPF and 500 ml of 0.9% sodium chloride was given 24 h after operation; mixed suspension that contains 500 ml TPF and 250 ml of 0.9% sodium chloride was given on the 3rd to 4th day after operation; 1000 ml TPF of total nutrient solution was given on the 5th day after operation and 1500 ml TPF of total nutrient solution was given from the 6th to 7th day; if energy supply is insufficient, intravenous infusion would be carried out according to the patient's post-dose reaction, and the measurement through the enteral nutrition tube is instilled intermittently from “less to more” (input for 4 h as well as intermittent for 30 min; the enteral nutrition tube was rinsed with physiological saline before infusion for fear of obstruction). This procedure is performed until the transition to a liquid diet at 9 : 00 am and 3 : 00 pm, the speed is gradually increased from 10 or 20 drops/min to 40 or 60 drops/min, and the temperature is controlled at 38 or 39°C.

*(2) Study Group*. Qihuang decoction, which contains *Astragalus membranaceus*, *Rheum officinale*, rhizome of largehead atractylodes, *Codonopsis pilosula*, Fructus aurantii immaturus, *Magnolia officinalis*, *Salvia miltiorrhiza*, and Radix Scutellariae was offered to instill. They were mixed according to the mass ratio 20 : 10 : 20 : 20 : 10 : 10 : 15 : 12 and the total weight of mixed medicine was 234 g. 500 ml H_2_O was added and boiled for 30 min according to [16]. The Crude Drug Decoction was filtrated and concentrated to 1.0 g/ml, and it was preserved at 4°C and rewarmed before administration. 150 ml was infused every time, the temperature was 38∼39, the speed was controlled at 30∼40 ml/min, and the total course of treatment was 7 days.

#### 2.4.3. Observation Indicators and Their Detection

3 ml venous blood was taken from the median vein of the elbow at 6 : 00 in the morning before surgery and 3, 7, and 14 days after surgery. HB was detected by automatic blood cell analyzer (xn-9000, Sysmex) using Kurt method; ALB, TP, and PA were detected by special protein analyzer (BN, Siemens) through BCG, biuret, and immunoturbidimetry; IgA, IgM, IgM, and CD3^+^, CD4^+^, and CD4^+^/CD8^+^ were detected by automatic chemiluminescence assay (AutoLumo Awoo Plus, Zhengzhou Antu Biological Limited Company) using immunoturbidimetry and flow cytometry. Statistics of complications during hospitalization after gastric resection included anastomotic leakage, abdominal hemorrhage, abdominal infection, pulmonary infection, incision infection, urinary infections, gastroparesis syndrome, and early mortality and gastrointestinal motor function recovery after the operation in gastric cancer such as time of bowel sound recovery, anal exhaust time, and defecation time.

Anastomotic leakage was diagnosed by various clinical manifestations such as fever, abdominal pain, and peritonitis. Gastric juice and bile intestinal contents can be seen in abdominal drainage and this confirms the diagnosis combined with digestive tract iodine angiography [18, 19]. Abdominal hemorrhage was defined when the progressive decrease of hemoglobin was more than 20 g/L in line with abdominal CT or color Doppler ultrasound [20]. Pulmonary infection was diagnosed when the body temperature > 37.5°C, white blood cell count >10 × 10^10^/L, and percentage of neutrophils >90% combined with chest X-ray or CT [21]. Abdominal, incision, and urinary infection was proved when postoperative bacterial culture is positive [22]. Gastroparesis syndrome was diagnosed by delayed gastric emptying ruling out no mechanical obstruction and gastric drainage daily more than 800 ml that lasts 10 days [23]. Death diagnosis is as follows: coma, brain reflexes, and apnea experiments show positive combined with an electrocardiogram [24]. Recovery time of bowel sounds, anal exhaust, and defecation was as follows: the time of onset of symptoms is recorded by the nurse and was given to the clinician in a written form, which used the time of record minus the end of surgery.

### 2.5. Statistical Analyses

Data analysis was performed using statistical software SPSS21.0 (SPSS Inc., Chicago, IL, USA). The measurement data was expressed as $\bar{x}\pm s$. When the data satisfies the normal distribution, the repeated measures analysis of variance was used. Otherwise, the Mann–Whitney *U* rank-sum test was used. The count data is expressed as a number of cases or as a percentage, and the comparison is checked by the Chi-square test or Fisher's exact test. *P* < 0.05 was considered to be statistically significant.

## 3. Results

### 3.1. Baseline Characteristics

The postoperative hospitalization of 237 patients with gastric cancer was analyzed. Among them, 120 patients were given postoperative basic data of Qihuang decoction combined with enteral nutrition and 117 patients with normal saline combined with enteral nutrition. Gender, mean age, tumor site, pathological stage, histological grading, surgical method, operative time, and intraoperative blood loss were recorded in Table 1.

### 3.2. Comparison of Nutritional Status in Both Groups

The ALB, TB, and PA in the two groups on Day 1 and Day 3 after operation were lower than those before the operation, and the decrease was most obvious after Day 1 (*P* < 0.05), but Days 7 and 14 after operation were significantly higher than those after Day 1 (*P* < 0.05). In the study group, ALB, TB, and PA 14 days after surgery were significantly higher than those before surgery (*P* < 0.05). There was no significant difference in HB, ALB, TB, and PA between the two groups 1 day before the operation. On Days 7 and 14 after surgery, the levels of ALB and TB in the study group were significantly higher than those in the control group (*P* < 0.05) and on Days 3, 7, and 14 after surgery, the levels of PA were significantly higher (*P* < 0.05). However, no statistical significance was found in HB between the two groups on Days 1, 3, 7, and 14 after surgery (*P* < 0.05) as shown in Table 2.

### 3.3. Comparison of Immune Function in Both Groups

CD3^+^, CD4^+^, CD4^+^/CD8^+^, IgA, IgM, and IgG in the two groups were significantly lower on Day 1 after the operation than those before the operation (*P* < 0.05), but the levels of CD3^+^, CD4^+^, CD4^+^/CD8, IgA, IgM, and IgG were significantly higher on Days 3, 7, and 14 after the operation than those on Day 1 after the operation (*P* < 0.05). Moreover, CD3^+^, CD4^+^, CD4^+^/CD8, IgA, IgM, and IgG in the study group exceeded the preoperative level on Day 14 after the operation (*P* < 0.05). Compared with the control group, IgA, IgM, and IgG increased significantly on Days 3, 7, and 14 after the surgery (*P* < 0.05), but CD3^+^, CD4^+^, and CD4^+^/CD8 increased significantly on Days 7 and 14 after surgery only (*P* < 0.05) as shown in Table 3.

### 3.4. Gastrointestinal Recovery and Complications in Both Groups

The rates of pulmonary infection, abdominal infection, incision infection, and urinary infection in the study group were significantly lower than those in the control group and the differences were statistically significant (*P* < 0.05). Although the rates of anastomotic leakage, abdominal hemorrhage, gastroparesis syndrome, and early mortality in the study group were lower than those in the control group, the difference was not statistically significant (*P* > 0.05) as shown in Table 4.

## 4. Discussion

Gastric cancer, the gastric epithelial malignant tumor, ranks fifth and second in death in the incidence of malignant tumors [25]. Its clinical manifestations are not specific. Early symptoms are only the upper abdomen being full of discomfort or dull pain, loss of appetite, and malignant vomiting and late symptoms include weight loss, fever, jaundice, and other cachexias, which are often ignored by patients. Therefore, active measures should be taken early in the clinical stage. Until now, surgery is still the main method of treatment for gastric cancer. However, partial gastrointestinal neurotomy and extensive resection of organs and tissues, as well as reconstruction of the digestive tract all together can lead to postoperative gastric motility and motor dysfunction by the disordering of gastrointestinal hormone secretion, so the patient's presentation is postoperative bloating, abdominal pain, and indigestion [26]. At the same time, surgical trauma, pathophysiological changes, and postoperative stress will also aggravate the catabolism of the body, resulting in malnutrition and immunosuppression [15, 27]. Malnutrition and low immune function lead to inflammatory reactions, which not only increase postoperative complications such as infection but also increase the recurrence of postoperative tumors and seriously affect the quality of life after surgery. Khorgami et al. found that immunosuppression and postoperative inflammatory response may lead to increased postoperative infection and tumor cell metastasis [28]. Esteban et al. have shown that the inflammation and immune status of gastric cancer patients are closely related to the occurrence of postoperative complications [29]. Therefore, the key to postoperative treatment is to select the appropriate nutritional support methods to improve the immune function of the body and correct malnutrition in time.

Early enteral nutrition has two advantages. Firstly, it can improve the recovery of intestinal peristaltic function by improving the height of intestinal villi, maintaining the mechanical barrier of the intestinal mucosa, protecting the growth of beneficial bacteria in the intestinal tract, and stimulating gastric acid secretion. Secondly, it is beneficial for liver protein synthesis and metabolism to promote early incision healing [30, 31]. Currently, the most popular ways are standard enteral nutrition and immune enteral nutrition. Some scholars believe that standard enteral nutrition support can correct the nutrition-related complications of patients, but the immune and inflammatory response is not obvious [15]. ESPEN (European Society of Parenteral Enteral Nutrition) recommends that patients with upper gastrointestinal tumors use immune enteral nutrition (glutamine, arginine, omega-3 fatty acids, and nucleotides) to promote lymphocyte proliferation and differentiation to improve immune function, shorten hospital stay, and control postoperative infection. However, whether immune intestinal nutrition is superior to standard enteral nutrition in terms of immune indicators remains controversial [27]. The research on the effects of traditional Chinese medicine combined with enteral nutrition support on the nutritional status and immune function of postoperative patients with gastric cancer has not been deepened. Previous animal experiments in our group have shown that Chinese herbal medicine of Qihuang decoction can not only improve the nutritional status of rats after gastrectomy but also increase the number of T cells and B cells in epithelial lymphocytes and lamina propria lymphocytes [32]. Our study provides a bold attempt to study the effects of Chinese herbal medicine of Qihuang decoction combined with EN on the nutritional and immune status of patients with postoperative gastric cancer.

Visceral protein is the most important nutritional monitoring index, including albumin, prealbumin, and total protein. Malnutrition has existed in the patients because of tumor consumption and tumor body release of toxins [33]. This study showed that the preoperative HB, ALB, TB, and PA were lower than normal. The surgical trauma, pathophysiological changes, and postoperative stress led to more catabolism in the body. The most significant decrease was also observed from our research on Day 1 after surgery (*P* < 0.05), which arrived at the same conclusion as they have. Prealbumin has a short half-life and good specificity, which can reflect the nutritional status and prognosis of patients [33]. The study showed that ALB and TB were significantly higher in the study group than those in the control group on Days 7 and 14 after surgery (*P* < 0.05), while PA in the study group was significantly higher than that in the control group on Days 3, 7, and 14 after surgery (*P* < 0.05). The analysis of nutritional status improvement reasons was as follows. On the one hand, gastrin, gastric acid, hormones, and enzymes can be more promoted in Qihuang decoction combined with enteral nutrition, which contributes to promoting the recovery of gastrointestinal motility and function and shortening the fasting time of patients. On the other hand, Qihuang decoction inhibits cell apoptosis and alleviates the injury caused by intestinal mucosal epithelium induced by ischemia reperfusion through upregulating Bcl-2 mRNA and downregulating the expressions of Bax, Caspase 3, and Caspase 9 mRNA, and this is more conducive to the absorption of intestinal nutrition [31, 34, 35]. Owing to the stress of surgery, intraoperative blood loss, and the inability to recover hematopoietic organs in a short time, the increase of HB is not obvious (*P* > 0.05).

Surgical stress induces neuroendocrine responses that promote the release of hormones such as catecholamine (norepinephrine and adrenaline), corticotrophin, and cortisol by activating the sympathetic nervous system and the hypothalamic-pituitary-adrenal HPA axis, which suppress the immune response [36]. Among them, CD3^+^ is a kind of T cells, which is also the basis of cellular immunity. CD4^+^ belongs to a kind of helper T cells. The importance of CD4^+^ cells in coordinating the immune response has increased significantly over the past decade. IgA, IgG, and IgM are mainly secreted by B lymphocytes to exert humoral immunity [37]. The body's immune surveillance is completed by T cells and B cells, reflecting the body's immune function and disease development. Yu et al. found that the postoperative cellular and humoral immunity of patients treated with Qihuang decoction increased significantly [38]. We found that the CD3^+^, CD4^+^, CD4^+^/CD8, IgA, IgM, and IgG in the study group exceeded the preoperative level on Day 14 after the operation (*P* < 0.05). We also found that IgA, IgM, and IgG in the study group increased significantly on Days 3, 7, and 14 after operation compared with the control group (*P* < 0.05), but CD3^+^, CD4^+^, and CD4^+^/CD8 increased significantly on Days 7 and 14 after surgery only (*P* < 0.05). At the same time, infectious incidences like pulmonary, abdominal, incision, and urinary system infection were also significantly reduced (*P* < 0.05). It further indicated that Qihuang decoction combined with EN reduced the incidence of complications significantly by promoting both cellular and humoral immunity, especially humoral immunity recovered earlier. Its possible mechanism is that Qihuang decoction controls inflammatory response and regulates immunity by inhibiting proinflammatory cytokines (IL-2*α*, IL-4, and IL-10) and upregulating anti-inflammatory cytokines (IL-1*α*, IL-6, and TNF-*α*). On the other hand, it has adjusted to the whole body humoral and cellular immune function, which could be explained with the effect site (mucosa lamina propria and intraepithelial) and parts of the sensitization collection of the lymphoid tissue (Peyer's patches (PP)). They mainly regulate the level of lymphocyte homing in the aggregated lymphoid tissue (PP) and mesenteric lymph nodes, so that the lymphocyte level in the blood rises to play the role of T lymphocytes and B lymphocytes [32, 39].

Therefore, our study showed that Qihuang decoction combined with EN could make up for the deficiency of EN alone. Astragalus polysaccharide can induce apoptosis of gastric cancer MGC-803 cells by blocking the S phase cell cycle and interfering with the mitochondrial intrinsic apoptotic pathway from modern pharmacological studies [40]. Atractylodes polysaccharide can promote the lymphocyte into the S phase and G2/M phase and increase the concentration of CD4^+^ and CD8^+^ in T lymphocytes and it is positively correlated with its concentration [41]. Rhubarb can enhance the innate immune homeostasis of the host mucosa by increasing the height of villi in the ileum, upregulating anti-inflammatory factor IL-10, reducing the proinflammatory factor IL-1*β* in the jejunum and ileum, and promoting the increase of claudin-1 mRNA and protein expression [42]. We in previous studies have confirmed that the fingerprint of Qihuang decoction has shared peak data information to ensure that there is no difference in the composition and efficacy in them where the main component of water-soluble saponin is baicalin. We have confirmed that baicalin enhanced the intestinal immune barrier mainly by promoting the proliferation and differentiation of intestinal mucosal lymphocytes and the synthesis and secretion of immunoglobulin by intestinal mucosal cells after gastrectomy in rat experiments. At the same time, the improvement of the mechanical barrier was also achieved by inhibiting the phosphorylation level of tight junction proteins [16].

## 5. Conclusions

Patients with gastric cancer after surgery are suffering from stress such as surgery and anesthesia, which can enhance the body's catabolism and lead to malnutrition and immunosuppression of patients that increase the risk of postoperative infection and other complications. Early treatment of gastric cancer after the treatment with Qihuang decoction combined with EN does not increase the incidence of complications such as anastomotic leakage and abdominal bleeding but reduces the incidence of infection. At the same time, it can accelerate the recovery of nutrition and immune function. We avoid abdominal distension and diarrhea caused by the infusion to adopt control of the infusion speed and temperature from less to more and slow to fast. Due to the limited sample size and selection of indicators this time, we will further refine the indicators and increase the sample size and further study the impact of Qihuang decoction on inflammatory indicators.

## Figures and Tables

**Table 1:** General information of patients with gastric cancer in the two groups.

Group	Study group (*n* = 120)	Control group (*n* = 117)	*t/χ* ^2^	*P*
*Gender*				
Male/female	81/39	96/21	3.087	0.079
Mean age(mean ± SD, years)	65.33 ± 11.41	66.75 ± 9.00	−1.070	0.286

*Tumor site*				
Cardiac region	12	10		
Fundus of stomach	28	25	0.936	0.817
Gastric body	38	34		
Antrum of stomach	42	48		

*Pathological stage(PTNM)*				
Stage I	32	28		
Stage II	42	39	0.717	0.869
Stage III	36	41		
Stage IV	10	9		

*Histological grading*				
Well	55	44		
Moderately	42	48	1.668	0.434
Poorly	23	25		
Surgical method				
Distal gastrectomy	38	33	0.338	0.561
Total gastrectomy	82	84		
Time of operation(min)	214.19 ± 8.24	213.29 ± 6.09	0.959	0.338
Intraoperative blood loss(ml)	220.06 ± 8.75	221.09 ± 7.76	−0.957	0.340

**Table 2:** Comparison of changes in nutritional status.

Indexs	Study group (*n* = 120)					Control group(*n* = 117)					*P * ^N^-value				
	Before	Day 1	Day 3	Day 7	Day 14	Before	Day 1	Day 3	Day7	Day14	Before	Day 1	Day 3	Day7	Day14
HB(g/L)	106.33 ± 7.47	98.31 ± 6.92^*∗*^	101.24 ± 7.00^*∗*#^	105.26 ± 6.87^#^&	107.26 ± 6.84^#&∆^	106.51 ± 11.14	97.52 ± 8.17^*∗*^	101.14 ± 15.59^*∗*^^#^	104.03 ± 10.19^*∗*^^#&^	106.19 ± 11.12^#^&^∆^	0.884	0.425	0.947	0.292	0.372
ALB(g/L)	35.90 ± 4.76	30.12 ± 4.55^*∗*^	32.14 ± 4.52^*∗*#^	36.23 ± 4.74^#&^	39.34 ± 4.58^*∗*^^#&∆^	35.15 ± 3.84	30.53 ± 4.73^*∗*^	31.45 ± 4.38^*∗*^^#^	33.87 ± 3.01^*∗*^^#&^	35.98 ± 3.61^#&∆^	0.185	0.499	0.237	≤0.001	≤0.001
TP(g/L)	58.23 ± 6.35	49.32 ± 5.73^*∗*^	52.35 ± 5.72^*∗*#^	57.60 ± 5.46^#&^	62.31 ± 5.26^*∗*^^#&∆^	59.41 ± 4.61	49.18 ± 6.96^*∗*^	51.61 ± 5.14^*∗*^^#^	52.88 ± 3.42^*∗*^^#&^	59.86 ± 5.98^#&∆^	0.103	0.872	0.301	≤0.001	≤0.001
PA(mg/L)	207.10 ± 32.74	201.32 ± 31.38^*∗*^	208.49 ± 24.79^#^	211.28 ± 19.33^*∗*^^#&^	213.30 ± 31.26^*∗*^^#&∆^	206.67 ± 21.30	200.97 ± 24.25^*∗*^	201.68 ± 14.41^*∗*^	205.12 ± 9.69^#&^	206.13 ± 12.45^#&∆^	0.775	0.925	0.011	0.002	0.022

(1) All values are means ± sem. There was no difference between groups before surgery. *P*^N^-value: study group compared with the control group. (2) ^*∗*^*P* < 0.05 compared with before surgery; ^#^*P* < 0.05 compared with Day 1; ^&^*P* < 0.05 compared with Day 3; ^Δ^*P* < 0.05 compared with Day 7 (all of them adopt ANOVA and LSD post hoc test) .(3) Normal range of these indexes: hemoglobin (HB): 130∼175 g/L; albumin (ALB): 40–55 g/L; total protein (TP): 60∼80 g/L; prealbumin protein (PA), 100–400 mg/L.

**Table 3:** Comparison of immune function.

	Study group (*n* = 120)	Control group (*n* = 117)	*P* ^*N*^-value
*CD3* ^*+*^ * (%) normal range: 60%∼80%*			
Before	50.11 ± 7.53	49.42 ± 5.37	0.419
Day 1	43.13 ± 7.64^*∗*^	42.67 ± 4.07^*∗*^	0.567
Day 3	47.34 ± 7.59^*∗*^^#^	46.06 ± 3.45^*∗*^^#^	0.097
Day 7	48.12 ± 7.32^*∗*^^#^	46.01 ± 7.25^*∗*^^#^	0.027
Day 14	52.20 ± 7.48^*∗*^^#&∆^	48.78 ± 7.06^#&∆^	≤0.001
*F-test*	4.069	*P* ^*M*^ * -value*	0.008

*CD4* ^*+*^ * (%) normal range: 35%∼55%*			
Before	29.00 ± 4.73	29.49 ± 4.22	0.404
Day 1	23.33 ± 3.64^*∗*^	24.42 ± 5.35^*∗*^	0.412
Day 3	27.01 ± 4.74^*∗*^^#^	26.43 ± 5.24^*∗*^^#^	0.370
Day 7	31.89 ± 4.09^*∗*^^#&^	30.05 ± 6.28^#^	0.008
Day 14	34.00 ± 4.66^*∗*^^#&∆^	30.64 ± 5.93^*∗*^^#^	≤0.001
*F-test*	9.990	*P* ^*M*^ * -value*	≤0.001

*CD4* ^*+*^ * /CD8* ^*+*^ * normal range: 1.4∼2.0*			
Before	0.79 ± 0.45	0.88 ± 0.27	0.098
Day 1	0.70 ± 0.40^*∗*^	0.75 ± 0.15^*∗*^	0.205
Day 3	0.82 ± 0.39^#^	0.80 ± 0.35^*∗*^^#^	0.731
Day 7	0.94 ± 0.38^*∗*^^#&^	0.84 ± 0.19^#^	0.010
Day 14	1.43 ± 0.64^*∗*^^#&∆^	1.01 ± 0.27^*∗*^^#&∆^	≤0.001
*F-test*	48.682	*P* ^*M*^ * -value*	≤0.001

*IgA(g/L) normal range:0.70∼4.06 g/L*			
Before	1.62 ± 0.38	1.59 ± 0.27	0.420
Day 1	1.32 ± 0.29^*∗*^	1.31 ± 0.16^*∗*^	0.592
Day 3	2.03 ± 0.53^*∗*^^#^	1.63 ± 0.36^#^	≤0.001
Day 7	2.45 ± 0.42^*∗*^^#&^	1.83 ± 0.19^*∗*^^#&^	≤0.001
Day 14	3.44 ± 0.42^*∗*^^#&∆^	2.67 ± 0.21^*∗*^^#&∆^	≤0.001
*F-test*	64.849	*P* ^*M*^ * -value*	≤0.001

*IgM(g/L) normal range:0.34∼2.14 g/L*			
Before	1.72 ± 0.36	1.66 ± 0.30	0.165
Day 1	1.10 ± 0.43^*∗*^	1.02 ± 0.25^*∗*^	0.079
Day 3	1.92 ± 0.39^*∗*^^#^	1.30 ± 0.44^*∗*^^#^	≤0.001
Day 7	2.30 ± 0.36^*∗*^^#&^	1.42 ± 0.22^*∗*^^#&^	≤0.001
Day 14	2.42 ± 0.41^*∗*^^#&∆^	1.90 ± 0.12^*∗*^^#&∆^	≤0.001
*F-test*	71.784	*P* ^*M*^ * -value*	≤0.001

*IgG(g/L) normal range:6.80∼14.50 g/L*			
Before	9.32 ± 3.86	9.64 ± 2.23	0.442
Day 1	6.30 ± 3.53^*∗*^	6.02 ± 0.32^*∗*^	0.394
Day 3	7.12 ± 3.38^*∗*^^#^	6.32 ± 1.51^*∗*^^#^	0.020
Day 7	8.35 ± 1.44^*∗*^^#&^	7.66 ± 0.40^*∗*^^#&^	≤0.001
Day 14	10.44 ± 3.31^*∗*^^#&∆^	8.92 ± 0.95^*∗*^^#&∆^	≤0.001
*F-test*	10.257	*P* ^*M*^ * -value*	≤0.001

(1) All values are means ± sem. There was no difference between groups before surgery. (2) *P*^*N*^*-*values for the difference between study group and control group with respect to the time point were calculated as treatment × time interaction. *P*^*M*^*-*values for the difference between study group and control group with all the time points were calculated as treatment × time interaction. (3) ^*∗*^*P* < 0.05 compared with before surgery; ^#^*P* < 0.05 compared with Day 1; ^&^*P* < 0.05 compared with Day 3; ^Δ^*P* < 0.05 compared with Day 7 (all of them adopt ANOVA and LSD post hoc test).

**Table 4:** Postoperative complications *n* (%) in the study group and control group.

Complication	Study group (*n* = 120)	Control group (*n* = 117)	Total (*n* = 237)	*χ* ^2^	*P*
Anastomotic leakage	4 (3.33)	6 (5.13)	10 (4.22)	—	*P* ^*f*^ *=* 0.359
Abdominal hemorrhage	10 (8.33)	13 (11.11)	23 (9.70)	0.522	*P* ^χ2^ *=* 0.470
Abdominal infection	5 (4.17)	15 (12.82)	20 (8.44)	5.742	*P* ^χ2^ *=* 0.017
Pulmonary infection	12 (10.00)	23 (19.66)	35 (14.77)	4.39	*P* ^χ2^ *=* 0.036
Incision infection	7 (5.83)	16 (13.68)	23 (9.70)	4.157	*P* ^χ2^ *=* 0.041
Urinary infection	5 (4.17)	18 (15.38)	15 (6.33)	8.507	*P* ^χ2^ *=* 0.004
Gastroparesis syndrome	2 (1.67)	5 (4.27)	7 (2.95)	—	*P* ^ *f*^ *=* 0.227
Early mortality	0 (0)	2 (1.71)	2 (0.84)	—	*P* ^ *f*^ *=* 0.243

(1) *P*^χ2^: in Pearson Chi-square test; *P*^ *f*^: in Fisher's exact test; *n*: the number of examined patients. (2) *χ*^2^: random variable Chi-square test value.

## Data Availability

The data used to support the findings of the study are available from the corresponding author upon request.
